# Destination Management in Times of Crisis - Potentials of Open Innovation Approach in the Context of COVID-19?

**DOI:** 10.1007/978-3-030-65785-7_49

**Published:** 2020-11-28

**Authors:** Markus Pillmayer, Nicolai Scherle, Katerina Volchek

**Affiliations:** 1 Department for Informatics, Technical University of Munich, Garching bei München, Bayern Germany; 2 Smart Tourism Education Platform (STEP) College of Hotel and Tourism Management, Kyung Hee University, Seoul, Korea (Republic of); 3 Department of Tourism and Service Management, MODUL University Vienna, Vienna, Wien Austria; 4 University of Applied Sciences Munich, Munich, Germany; 5 FOM Hochschule Für Oekonomie and Management, München, Germany; 6 Deggendorf Institute of Technology, Pfarrkirchen, Germany

**Keywords:** Open innovation, Destination management, Coronavirus, COVID-19, Innovation management, Smart destination ecosystem

## Abstract

The COVID-19 pandemic has led the tourist industry to a standstill. It also creates the potential to change both the global tourism industry and the context in which innovation management takes place in the medium to long term. The web-based open innovation approach provides one of the possibilities to uncover technical opportunities in the context of rapidly changing environment. This article takes a qualitative approach to explore the benefits of open innovation approach for destination management organizations (DMOs). The analysis of 15 semi-structured interviews reveals nine specific benefits that arise from the three types of open innovation. The findings deepen an understanding of the potential that the open innovation approach offers for DMOs. The study further creates a background to collaborative tourist recovery COVID-19 pandemic through smart destination ecosystems.

## Introduction

The coronavirus pandemic (COVID-19) has triggered an unprecedented crisis in the global tourism industry, given its immediate and immense impact. The tourism industry is one of the sectors most directly affected by the current crisis, particularly due to the fact that international air traffic has virtually ground to a halt [1]. De facto, travel, and everyday tourism is still far from the pre-Corona phase. The changes affect almost every aspect of the ecosystem of destination management organizations (DMOs). DMOs are need new and innovative formats in the course of this paradigm shift in order to gain competitive advantages. In the context of global competition it has become a condition sine qua non for destinations to distinguish themselves decidedly from competitors in order to successively and sustainably compete on the travel market [2].

A range of technological innovations and digital solutions of smart destination ecosystems are becoming available for DMOs [3]. Digitization and smart environments create a borderless, international competitive environment for the tourism industry [4, 5]. Innovative formats create new opportunities for tourism service development and process optimization in general and DMOs in particular [6]. However, their application often requires paradigmatic changes in destination development strategies [7, 8]. Thus, it becomes necessary to involve a large number of stakeholders, and opening up to an innovative solution for DMOs’ strategy in order to benefit from such technologies [4, 9].

The concept of open innovation explains the logic of engaging multiple stakeholders in an innovation process. Technology-oriented organizations can benefit from engaging multiple stakeholders at different stages of the innovation process. An appropriate combination of actors, their resources, including knowledge and technology, and institutional arrangements, can facilitate value co-creation in smart tourism ecosystems [10].

Among others, application of open innovation allows organizations to acquire missing resources and optimize internal processes of the organization [11]. There is an evidence that technology-driven open innovation has potential for strengthening competitive position of organizations. Sustainable development of businesses can be achieved through new collaboration, funding opportunities, intensified engagement, increased diversity and supported infrastructure [12]. However, the potential for changing smart tourism destinations to create value by engaging multiple stakeholders remains underexplored.

The following study aims to explore the benefits of applying open innovation strategy by destinations. It first defines an open innovation strategy and conceptualizes its three major types in relation to the objective of resource exchange processes between organizational and external stakeholders. The study then explains the applied qualitative research design and reports the findings of the 15 semi-structured interviews with the DMOs’ experts. The study identifies 9 key benefits of open innovation strategy and increased involvement of tourists, locals, and other stakeholders, for tourism destinations. The study contributes to the domains of destination management and service innovation. It provides insights of successful implementation of open innovation in the context of global competition, developing smart ecosystems, and the pandemic. A discussion, which is primarily intended as a plea for the use of open innovation in the destination context, rounds off the article.

## Theoretical Background: Open Innovation

Innovations play an important role in the development of increasingly fragmented and pluralistic societies. They are seen as drivers of economic development, which offer an opportunity for individual companies to remain competitive [13]. With regard to tourism, innovations are acknowledged to be a driving force for the entire tourism industry, especially in times of a crisis [14, 15]. Though, the degree of change an innovation can bring largely depends on the type of engaged transformational processes [16].

It is common to distinguish between close and open innovations. Closed innovation refers to the innovation process with all stages being performed within the boundaries of an organization [15]. Organizations traditionally carry out each step from the generation of ideas to the actual development of an innovative solution, its market launch, and distribution, on their own and with their own resources. The closed innovation concept is contrasted with open innovation. Chesbrough [17] defines an open innovation as “…the use of purposive inflows and outflows of knowledge to accelerate internal innovation, and expand the markets for external use of innovation, respectively.” Organizations could take an advantage of both internal and external resources, including new ideas about the innovative solution, resources for its implementation, and consequent positioning on the market [18]. The tasks of an organization’s R&D department are, therefore, no longer limited to generating ideas themselves and developing them further. Instead, it is a matter of integrating new, external knowledge and expertise into the organization. It also becomes possible to share those ideas, generated within the organization, externally, or to combine internal and external resources [16]. The nature of innovation processes and the origin of resources, play a role in enabling the benefits of innovation strategy.

Depending on the nature of innovation processes and the origin of resources, it is common to distinguish between 3 different types of innovation models [9, 19]. An outside-in process allows to innovate by integrating the resources of external stakeholders. This may include external networking, integration of suppliers or customers in the design process, benefiting from external funds. An outside-in process can lead to an increase in the innovation capacity of the organization [11]. An inside-out process makes an organizational resource, including intellectual property and self-developed technologies, available to external stakeholders. This might refer to selling the result of research or technology development, sharing ideas with markets, or granting licenses. Lastly, the coupled process represents a combination of outside-in and inside-out processes. It can be achieved through strategic alliances with complementary partners, through joint ventures or co-patenting [19]. Hjalager & Nordin [20] give an overview of 16 practices of integrating external stakeholders into the innovation process. However, it is often difficult clearly to differentiate between the three types of open innovation [21]. The choice of the innovation process type, therefore, depends on the objective of engagement with external stakeholders.

Open innovation strategy facilitates new ways of meeting the abovenamed challenges with confidence. It allows to track down unknown sources of knowledge, and constructively integrate them into the innovation process [22]. First, it creates the opportunity to use external technologies and ideas than to generate them themselves and make them available to the ecosystem [23]. For DMOs, the open innovation strategy offers potential for aggregating ideas and using them as a starting point for the internal development of tourism services [24]. Accordingly, the added value of open innovation for destinations lies in acquisition of external knowledge [15]. Second, the open innovation strategy allows to intensify active participation of all interested stakeholders and enables them to participate in the design, development, and ultimately implementation of their “own” tourism service or product. This reduces costs and significantly increases fit-to-market. Third, open innovation and collaboration active collaboration between internal and external stakeholders inform them about each other, supporting marketing, the formation of a positive brand image, and the commitment to the DMOs [25–27]. Meeting the objectives of intensifying mutually beneficial collaboration, acquisition of new ideas and marketing, can be helpful for destinations in gaining a competitive advantage [18].

The benefits that the open innovation approach may provide, have triggered a paradigmatic change in destination management. More DMOs turn from the conventional closed innovation process, which has been popular in the 20th century, towards an open innovation process. Thus, a range of DMOs, including the Vienna Tourism Board, Tirol Werbung, Salzburg State Board of Tourism, and Swiss Tourism Board, called for open collaboration to generate ideas to increase the destinations’ competitiveness, revitalize tourism after the crisis while ensuring sustainable development of the regions [28–31]. However, the strategy of open innovation still lacks a comprehensive explanation in relation to benefits it can bring to a DMO [32]. Potentially, this prevents effective decision-making towards a specifics type of open innovation strategy and its efficient implementation.

## Methodology

This study aims to identify the benefits of applying open innovation strategy by destinations. It applied the discussed types of open innovation as a framework to inform the research design and searched for common benefits of open innovation strategy. Since open innovation is still a relatively young research phenomenon in destination context, the study accepted a qualitative respectively exploratory research design [33].

Qualitative research must comply with the methodological principles of openness, flexibility, communication, reflexivity and explication. Furthermore, the process of research and subject under investigation should be considered [34–36]. In doing so, qualitative studies revolve around the question of what interviewees consider relevant, how they observe their world, and what characterises their respective lives. In this context, it is particularly important to understand what makes people act in a certain way in a social context, what dynamics this action triggers in the social environment and how this affects the way they (re-)act. Against this background, this qualitative study focused on societal anchoring of practice of human actions, social events and their developmental dynamics with the view attempt to feed this into a theorising understanding. Qualitative research was done to procure the materials that meet the above named requirements.

The data acquisition process was regarded as a communicative achievement in which the corresponding colloquial utterances and actions were interpreted based on understanding of the overall context of the research situation [37]. The study applied a convenience sampling approach to access the total number of 15 destination management experts with the relevant expertise [38] in the period 2018 to 2020. 13 of them were DMO executives from Germany, Austria, and Switzerland. 2 interviewees were the leading experts in the field of open innovation and worked as consultants in the tourism field. All of them have proven international experience and have completed open innovation projects. The contact to the interview partners was established via a snowball technique. The interviews lasted between 45 and 90 min and were conducted both in the offices of the interview partners and, with the beginning of the COVID-19 pandemic, via Skype. The interviews were conducted and transcribed in German. To ensure data validity, a back-translation to English, made by the interpreters with the relevant expertise, was conducted.

The research used semi-structured individual interviews to asked the experts to explain their motives of carrying out an open innovation project. Additionally, their perceptions of the benefits that the DMOs have acquired have been collected. The questions used in the present study were adopted from the study by Storch and Pillmayer [2]. The study then applied thematic coding principle to analyze the English version of the interviews. The themes were deductively derived from the conducted interviews. The coding was done with MAXQDA software, which is proven to be a reliable tool to ensure systematic content analysis of the tourism science context [2]. The identified themes were used to explore the common trends in the practices.

## Findings: The Benefits of Open Innovation for Destinations

This section reports the results of the qualitative content analysis. Overall, most of the experts agree that the objective of applying the open innovation strategy lies in obtaining new knowledge and creative inspiration in the sense of thinking outside the box, especially in times of crisis. A crisis offers a paradigmatic opportunity to critically question everyday rituals and practices and to develop them further using suitable instruments. This aspect becomes especially important in the context of unexpected restrictions and new regulations that must be observed in the context of COVID-19. The impact of open innovation on external stakeholders including marketing and brand image plays a central role in the DMOs strategy. Active engagement of a large number of internal and external stakeholders is also confirmed to the advantageous for achieving the aim of the DMOs and the external stakeholders.

**Fig. 1.**
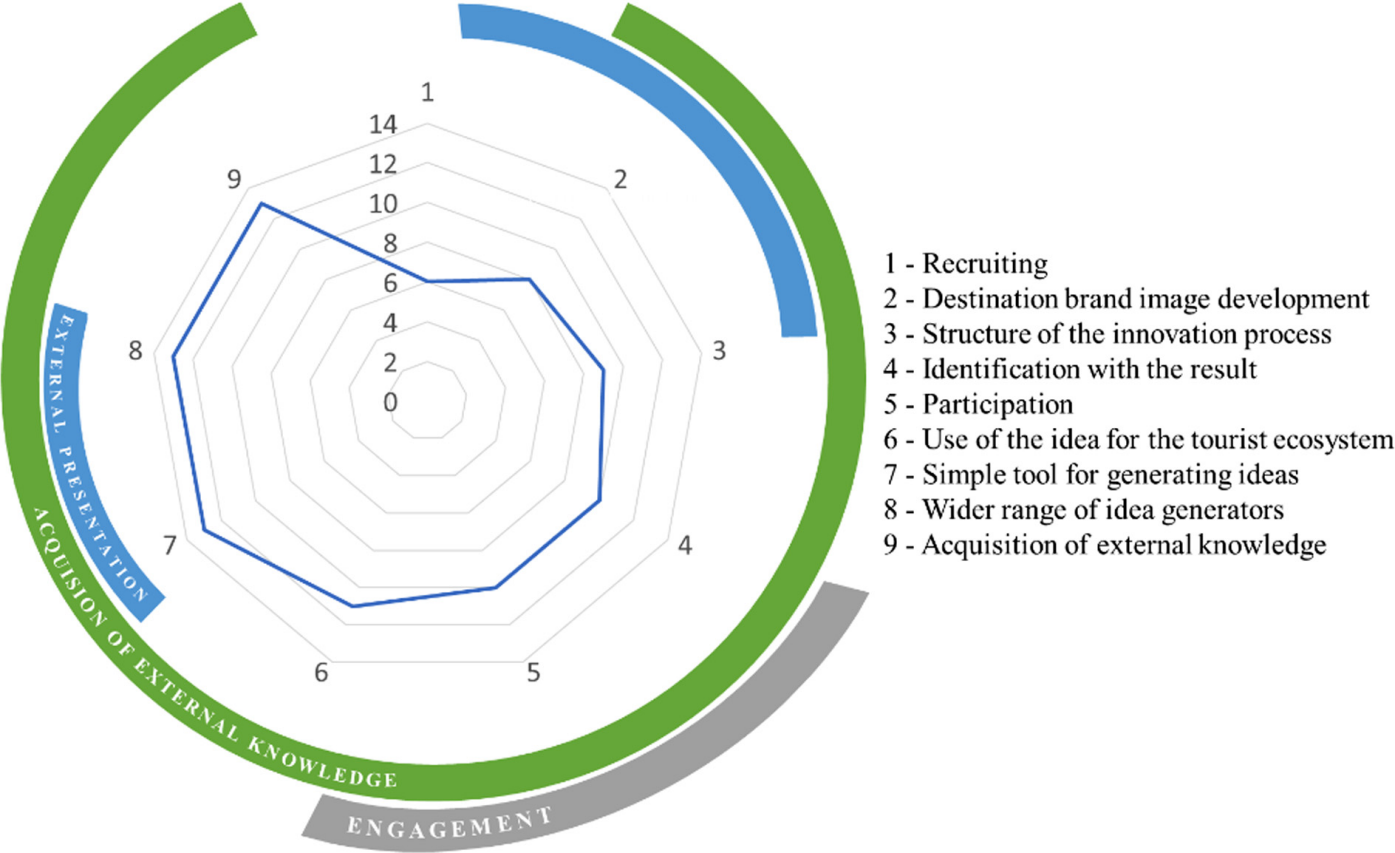
Benefits of Open Innovation for DMOs

The study has identified a total of 9 benefits of applying open innovation approach by DMOs. Figure 1 summarizes all of them with the reference to the number of responses among the experts. It further demonstrates that each of the identified benefits can contribute to more than one major objective of applying the open innovation strategy. The paper now proceeds with explaining those benefits within each of the three major objectives.

### Acquisition of New Knowledge for Destination Development

The respondents see open innovation as an opportunity to gather external impulses and actively involve third parties in the development process. It is explained as a simple tool for generating new know-how and ideas (Fig. 1, Aspects 7, 8, 9). Accordingly, the aim of a DMOS is to obtain input from others and, above all, new input. This can be achieved by involving a larger circle of idea providers, who may not necessarily have tourism expertise, but who can provide new and possibly unconventional ideas. Several interviewees see great potential in involving people who are not rooted in the destination’s ecosystem (Fig. 1, Aspect 6), but come from other sectors. The project manager of an open innovation project, which was one of the most successful tourism projects in Europe in 2018, sums up the added value in a nutshell:

*“With open innovation, you broaden your horizon and come up with completely different ideas and possible solutions. That can also work well. With the limitations of meeting other people, this approach works quite well. I think that the strength of open innovation is diversity, on the one hand. That people with different backgrounds, different experiences, different knowledge contribute to their different skills. On the other hand, of course, the fact that I can contribute from my own living room at home via such a platform.”*

On the other hand, one hopes to receive more detailed ideas that more precisely meet the needs of the stakeholders (Fig. 1, Aspect 4). The consultant of one of the leading tourism consulting agencies in the German-speaking world, states:

*“The decisive advantage is to get more ideas, more approaches, and more precise and detailed information about what exactly the clients and stakeholders actually want. To gain a deeper insight into their needs. To me, open innovation is basically about managing cross-border knowledge flows purposefully. Due to COVID-19, we cannot reach the customer in a conventional way. Clearly, that is a disadvantage. It’s a different story with a digital solution where the customers feel that they are being taken along.”*

According to the experts, the involvement of tourists is a key added value for the development of innovations (Fig. 1, Aspects 2, 5). It enables destination management to gain the insights of those individual tourist needs, which they might not have articulated in a conventional way of customer feedback. One expert describes that open innovation offers their organization a good opportunity to involve customers on a large scale (Fig. 1, Aspect 2). They summarize the resulting added value as follows:

*“What was positive in any case was that you can see what is important to the customer, especially with regard to hygiene and health standards. There was a lot there, we didn’t even think about it because we are of course also in our bubble. When things are mentioned ten or twenty times, you notice that you have to look at them or it is important to address them. There were some good considerations.”*

Although some of the interviewees point out that many of the generated ideas may have been “not surprising”, they emphasize that tourists’ ideas and perceptions about their wishes are a great benefit for their organizations. Innovations that produce personalized services, tailored to specific needs of the target groups, increase the fit-to-market and strengthens the organization’s competitiveness (Fig. 1, Aspects 2, 3).

In summary, the experts see the added value of open innovation in the acquisition of external ideas. They conceptualize open innovation as a proven opportunity to involve a broader group of idea providers. In particular, they emphasize the involvement of guests and industry outsiders. The definition of the basic types of open innovation [9] show that open innovation is a process in which knowledge can diffuse in two directions. In particular, when open innovation has been used in the context of strategy development, the interlocutors can focus on further development of the ideas gained outside their own organization. The experts describe how open innovation strategy development gives stakeholders impulses for new projects, especially in the area of processes, services, and products, and how they can pursue these independently.

### External Presentation of Destinations

The study reconfirms that open innovation projects are implemented with the objective of marketing destinations [39]. The tourism experts see open innovation as an opportunity for a positive public image of the destinations, especially in order to clearly differentiate themselves from competitors (Fig. 1, Aspects 2, 7, 8). The employee of a state tourism marketing organization emphasizes the benefits of an open innovation project for the external image of a destination:

*“I think that a relatively large marketing effect is involved. Firstly, the product we have created through open innovation introduces our destination and its various offers. In this way, we draw attention to what the region has to offer and sensitize to confidence-building measures. The Swiss colleagues have done this quite well with the Clean & Safe campaign, for example!”.*

According to the experts, a meaningful combination of an open innovation process with a marketing campaign can increase attention to one’s own organization and its products. This, in turn, can raise the overall awareness of a destination (Fig. 1, Aspects 2, 3). A destination receives an opportunity to present itself as an authentic unit, which is open to concerns of existing guests and potential new customers. However, a destination and its individual service providers do not present themselves solely to their guests and new customers. Simultaneously, they are targeting future potential employees, especially in times of a shortage of skilled workers (Fig. 1, Aspect 1). Those service providers, who proactively involve their current or new employees, gain a strategic advantage [40, 41]. The innovation manager of one of the organizations also sees it this way:

*“At first glance, a mountain railway is not necessarily interesting for future employees, especially against the background of various doubts about climate change. We, therefore, launched an open innovation platform as part of a recruiting process and called on young people to think about how they would like to make our mountain railway operation sustainable. What were their ideas to keep it running in the next 10, 20, 30 years? Of course, this resulted in some crazy things, but we were able to see who had the analytical know-how and who was the best match for us in terms of mindset.”*

Apart from the attention, that such an unusual initiative attracts (Fig. 1, Aspect 2), open innovation can help DMOs to connect with young professionals. By using the means of an open innovation platform, your people can address service providers in the tourism value chain and present themselves in a possibly creative way. The potential employer can check apart from conventional procedures such as interviews or assessment centers to see who is best suited to the company in the medium to long term.

### Increased Degree of Engagement of Influential External Stakeholders

Another positive aspect, which is particularly emphasized by the participants in strategy development processes, is the degree of engagement that can be achieved through the open innovation process (Fig. 1, Aspect 5). In this context, the increasing appreciation of a product goes hand in hand with the participation of external stakeholders [18]. With the help of open innovation, a large number of stakeholders could be involved in tourism projects (Fig. 1, Aspect 6). This, in turn, is helpful for optimizing stakeholder management, as the strategy manager of a state tourism organization points out:

*“I see it as an opportunity to make tourism more inclusive again. How to enter into a discourse with all those who really participate in tourism and make a contribution to tourism. And how to present comprehensibility of the significance of what we are doing here, especially to many a critic. Because to me, that is the biggest challenge, even within the destination. That way everyone can get involved, everyone feels taken along.”*

Even though this aspect may not be directly aimed at creation of innovations, it does generate added value for the destinations. According to the interviewees, this happens due to the selected open innovation strategy. As a result of the high level of participation in the strategy development process, the experts describe that the outcome of an innovation is strongly identified with the participation of the stakeholders within destinations (Fig. 1, Aspects 4, 5, 6). External stakeholders are behind the strategy and show more commitment to the implementation of the individual points. One of the interviewees summarizes the advantages that the open innovation strategy development offers to a region:

*“So far, we have not done this in tourism, involving people on such a broad scale. At the beginning, people were quite critical of our project and distanced themselves from it. But it has now become a consequence, especially in terms of implementation: because we notice that the people we once involved want to continue to be involved. They really liked it, they thought that now we are being asked and that we must continue to work on it, that is our baby!”.*

It turns out that the involves external stakeholders affect the destination innovation strategies along the whole process rather than only at the development stage. Thus, a higher level of commitment can lead to increased loyalty to a product. The head of a development department for open innovation projects describes:

*“[The DMO] had the opportunity to actively involve guests and locals in the ski design process. The participants could choose and decide which one was the winning design. Specifically, cards with the designs of the three finalists were printed. The participants could mark their favorite design and send the card. So, the DMO had the opportunity to actively involve the participants and thus close this cycle. They could vote and decide which is the winning design and next season they had the opportunity to rent this ski in the ski rental and then ski with it on the slope themselves. This creates a completely different bond with my customers than would otherwise be possible. That’s what I see as the decisive added value.”*

This statement in particular makes it almost paradigmatically clear, how co-design and the associated customer loyalty to the tourist product are realized. Locals and guests subsume the process as “their own product”, which again gives it a completely new perspective on the tourist experience.

It becomes clear that the identified 9 benefits are interdependent. Each of them can influence the successful realization of several objectives that serve the reason for selecting the open innovation strategy over the closed one. Therefore, comprehensive planning to optimize the effect of the open innovation strategy for DMOs is required.

## Discussion: The Potential of Innovation Management in Destinations?

Even though various industries have been making use of open innovation for some time now, the tourism industry still faces certain challenges in its application. The general tendency of a constantly changing complex competitive environment and increasingly divergent guest demands affect destinations. DMOs will face the need to open up to new options in order to co-create innovations. For the time being, DMOs often focus on short-term goals, including operational business and stake-holder management. In the context of constant time pressure and high-risk aversion, this leaves little time to address long-term strategic issues such as innovation management and related approaches. The pandemic revealed the unreadiness of DMOs to ensure competitiveness and sustainable collaboration between their stake-holders.

This study reconfirmed that open innovation strategy offers new opportunities for DMOs. This becomes especially important in times when the traditional dialogue with guests, locals, and stakeholders, loses its value. First, the open innovation approach creates an opportunity to generate ideas with the help of external knowledge. Chances lie in looking beyond the horizon and involving a larger circle of idea providers. External stakeholder, who are not rooted in the familiar ecosystem of the destination or DMOs, who have no expert knowledge, who come from other industries, and who, through their involvement, can raise completely new and sometimes, unusual aspects. Second, open innovations can lead to collaborative development of tourist services that are better tailored to the actual tourist needs of the respective target group. This ultimately increases the fit-to-market, especially against the background of sensitive issues in the context of hygiene and health. Furthermore, the targeted involvement of customers can increase customer loyalty to the destination and create positive marketing effects. Third, open innovation can be an effective means of involving more stakeholders. Increased involvement also means that stakeholders identify much more strongly with the outcome of the process and thus show more commitment in implementing the individual considerations. This is beneficial for both the guests, the stakeholders within a destination, and ultimately for DMOs. Importantly, the findings demonstrate that many of the benefits, derived from the open innovation approach, can contribute to several different objectives of applying open innovation approach.

The proliferation of smart tourism destinations may create a basis to boost open innovation approach. Smart destinations represent ecosystems of multiple players, including businesses, individuals and technology [3, 4]. In addition to interconnectivity and interoperability of devices, smart destinations rely on mutually-beneficial collaboration between multiple stakeholders [4]. In this case, incorporating the open innovation approach in smart destinations may inform the logic of collaboration processes: outside-in, inside-out or coupled [42]. Open innovation approach may also inform smart destination planning by naming the possible benefits that may arise. Simultaneously, smart destinations may boost open innovation by enabling technical part. Thus, interconnectivity and interoperability enable multiple actors to contribute to DMOs with data, knowledge, ideas and other resources in order to co-create value [4]. Service personalization, which aims to tailor services according to individual real-time tourist needs and deliver it in the way and time, that are relevant for the target customer, is named among the revolutionary outcomes of smart destinations [43]. Smart infrastructure and established networks of smart destinations may serve as a background to intensify collaboration and mutual engagement of local community and tourists in a DMO and vice versa [44]. Importantly, the focus of technology-driven open innovation for DMOs partially aligns with the one, applied by travel businesses, which, among others, also aim to intensify collaboration and engagement between stakeholders and support the development of infrastructure [12]. However, the concept and technical solutions of DMOs has its specifics in comparison to other actors of smart tourism ecosystems. Moreover, they are constantly evolving, sometimes providing unexpected outcomes. Since open innovation projects nevertheless involve considerable effort, they must be strategically planned and professionally implemented.

Each DMO must individually weigh up the advantages and disadvantages of the approach in order to decide for itself whether it wants to take this path. One of the interviews consultants for open innovation highlights:

*“There is no such thing as ‘one size fits all’. It depends on the innovation challenge or the area in which I want to innovate. There is no ‘all-purpose solution’ for this. It has to be clear to everyone involved.”*

Furthermore, the necessary mindset, associated with open innovation, often reaches its limits, as some stakeholders in destinations with resentment towards unusual approaches remain at a critical distance. The central challenge will be to break down and overcome these boundaries in order to raise awareness of the necessary innovations among destination stakeholders and to successfully exploit the opportunities offered by open innovation for destinations.

## Conclusion

This paper explored the benefits of applying open innovation approach by destinations. It has identified a total of 9 benefits that arise for DMOs from this approach. The findings provide the evidence that these benefits enable destinations to meet at least one of the three key objectives of applying the open innovation approach, namely, to access external knowledge, to support the presentation of the brand externally, and to intensify engagement among key internal and external stakeholders of a destination.

The study contributes to the domain of destination management. The findings align with the more generalized concept of open innovation [9]. They also demonstrate that the technology-driven open innovation strategies for DMOs may partially align with those, implemented by tourism businesses [12]. The developed context-specific insights deepen understanding of the potential of open innovation approach for DMOs and create a background to collaborative tourist recovery COVID-19 pandemic through smart destinations ecosystems. The study raises the question to what extent an open innovation and involvement of multiple stakeholders can represent an approach that develops tailor-made products for an international competitive environment, sustainably strengthens the competitive position in the international competition of destinations, and supports engagement with local communities through smart destination ecosystems. As a result, the findings provide conceptual background to the basic understanding of open innovation for destination management, including planned development of smart destinations and crisis management in the times.

The main limitation of the exploratory nature that this study took is the focus purely on the positive aspect of the open innovation strategy. Innovation in general and the incorporation of multiple stakeholders, who can influence the outcome of organizational activities, strips the organizations of control and increases the risks of failure [11]. Future research will focus on a deeper exploration of the open innovation strategy in one system with the threats, posed by it. Moreover, a roadmap of successful theory-driven practices on the incorporation of the open innovation strategy is also required.
